# Improvement in Chest Pain Following Surgical Treatment for Thoracic Outlet Syndrome

**DOI:** 10.3400/avd.cr.25-00043

**Published:** 2025-08-30

**Authors:** Shutaro Makita, Taku Suzuki, Yasuhiro Kiyota, Noboru Matsumura, Takuji Iwamoto, Masaya Nakamura

**Affiliations:** Department of Orthopaedic Surgery, Keio University School of Medicine, Tokyo, Japan

**Keywords:** chest pain, endoscopic-assisted infraclavicular approach, thoracic outlet syndrome

## Abstract

A 41-year-old woman with a 1-year history of right chest pain, with normal cardiology and pulmonology assessments. The chest pain was reproducible upon upper limb elevation. Computed tomography (CT) angiography in the arm-elevated position revealed subclavian artery and vein stenosis at the costoclavicular space, and the diagnosis was neurogenic thoracic outlet syndrome (TOS). Surgery involving endoscopic-assisted infraclavicular resection of the first rib and scalene muscles resulted in immediate postoperative symptom improvement. When chest pain persists after ruling out other conditions, neurogenic TOS should be considered in the differential diagnosis.

## Introduction

Thoracic outlet syndrome (TOS) arises from the compression of neurovascular structures within the thoracic outlet.^1)^ The primary symptoms typically include numbness or pain in the upper limb; however, autonomic dysfunction- related symptoms such as headaches and dizziness have also been reported.^1,2)^ Although chest pain has been documented as an associated symptom of TOS,^1)^ few reports describe it as the primary complaint. This case report highlights a patient with TOS whose chief complaint was chest pain, which resolved following surgical intervention. Written informed consent was obtained from the patient, and the study was approved by the ethics committee of our institution (Approval No. 20130147).

## Case Report

A 41-year-old woman presented with right chest pain for the past year. She consulted the departments of cardiology and pulmonology, but no abnormalities were detected on electrocardiography, echocardiography, chest X-ray, or computed tomography (CT). The patient’s symptoms did not improve, and she was referred to our hospital.

**Fig. 1:**
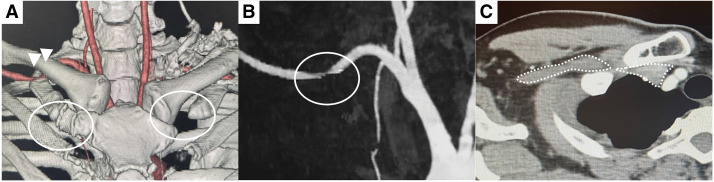
Computed tomography angiography in the arm-elevated position. (**A**, **B**) Asymmetry of the first ribs and stenosis of the subclavian artery at the costoclavicular space. (**C**) Stenosis of the subclavian vein at the costoclavicular space.

The patient reported chest pain localized at the right third and fourth ribs at rest. Tenderness was observed beneath the clavicle, and percussion of the supraclavicular fossa elicited radiating pain in the chest wall. The Roos test (90° shoulder abduction and external rotation) exacerbated the pain, and the hyperabduction (Wright) test yielded a positive result. The pain was reproducible with upper limb elevation. Although upper limb numbness and pain were induced by arm elevation, these symptoms were very mild. Cervical spine radiography and 3-dimensional CT revealed asymmetry of the first ribs: the right rib was ossified anteriorly, whereas the left rib exhibited anterior cartilaginous formation or hypoplasia (**Fig. 1A**). Cervical spine magnetic resonance imaging findings were normal. CT angiography in the arm-elevated position demonstrated subclavian artery and vein stenosis at the costoclavicular space (**Figs. 1B** and **1C**). Based on the clinical and imaging findings, the patient was diagnosed with neurogenic TOS and underwent endoscopic-assisted resection of the first rib and scalene muscles.

**Fig. 2:**
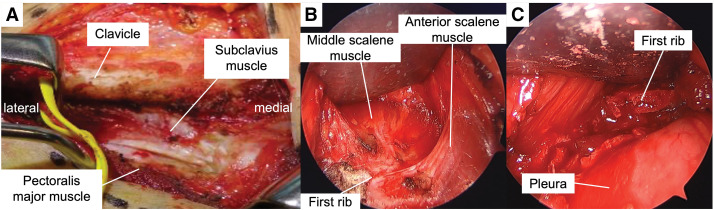
Operative findings. (**A**) The pectoralis major was released from the clavicle to expose the subclavius muscle. (**B**) Endoscopic view showing the first rib and scalene muscles. (**C**) Resection of the first rib.

**Fig. 3:**
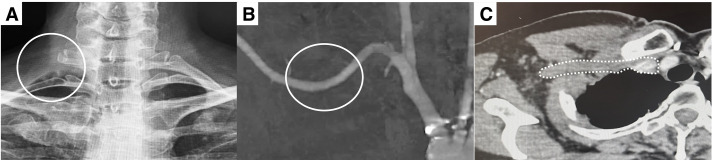
Postoperative images. (**A**) Radiograph of the first rib. (**B**) Computed tomography with the arm elevated showing improvement of subclavian artery stenosis at the costoclavicular space. (**C**) Computed tomography showing improvement of subclavian vein stenosis at the costoclavicular space.

A 7-cm transverse incision was made 1 cm below the clavicle, preserving the supraclavicular nerves. The pectoralis major muscle was released from the clavicle, exposing the subclavius muscle, which was subsequently excised (**Fig. 2A**). The subclavian vein and first rib were identified, and the vein was dissected to create a space between it and the rib. The subclavian vein, subclavian artery, and brachial plexus were protected using a muscle retractor. A 4-mm, 30° oblique endoscope (1488 HD Camera System; Stryker, Kalamazoo, MI, USA) was inserted through the same incision, providing good visualization of the anterior and middle scalene muscles as well as the posterior aspect of the first rib (**Fig. 2B**). These muscles were detached from the first rib using electrocautery. The first rib was carefully separated from the pleura and resected in sections with an endoscope-assisted rongeur to prevent pleural injury (**Fig. 2C**). Using the endoscope, the rib was excised near the transverse process of the T1 vertebra (**Fig. 3A**), and the pectoralis major was sutured back to the clavicle.

No postoperative complications, including pneumothorax, hemothorax, neurovascular injury, or hematoma, were observed. The patient’s preoperative chest pain resolved within 1 month after surgery. Two months postoperatively, CT angiography with the arm elevated showed improvement in subclavian artery and vein stenosis (**Figs. 3B** and **3C**). At the 24-month follow-up, the patient remained asymptomatic, with no recurrent chest pain or upper limb numbness upon elevation.

## Discussion

This case highlights TOS presenting primarily as chest pain, which resolved following first rib and scalene muscle resection.

The exact mechanism underlying chest pain in TOS remains unclear; however, potential contributors include the medial and lateral pectoral nerves, as well as the subclavius nerve, which originate from the brachial plexus.^3–5)^ The lateral pectoral nerve arises from the lateral cord of the brachial plexus, containing fibers from C5 to C7,^6)^ whereas the medial pectoral nerve originates from the medial cord, comprising fibers from C8 and T1.^6)^ These motor nerves innervate the pectoralis major and minor muscles, yet have been reported to possess sensory components capable of generating anterior chest pain.^5)^ Notably, pectoral nerve blocks have been shown to reduce opioid use in mastectomy surgeries.^4)^ Ferro et al. reported that lateral pectoral nerve injury can induce pectoral pain, which was effectively treated with ultrasound-guided pulsed radiofrequency therapy.^5)^ These findings suggest that pectoral nerves derived from the brachial plexus may contribute to chest pain in TOS. In cases with bony anomalies, as observed in this patient, abnormal ligaments or fibrous bands may compress neurovascular structures, potentially entrapping the pectoral nerve.^7)^ Pectoral nerve blocks may aid in diagnosing chest pain associated with TOS.

Surgical approaches for TOS include transaxillary and supraclavicular techniques.^8)^ However, these approaches pose challenges when addressing anterior thoracic outlet structures due to the position of the subclavian vein.^8)^ The infraclavicular approach is not considered to provide good surgical visibility in the posterior surgical maneuver due to the narrow space.^8)^ Recently, an endoscopic-assisted infraclavicular approach has been introduced for first rib and scalene muscle resection in neurogenic TOS.^9,10)^ This technique facilitates subclavian vein release, anterior first rib resection, subclavius muscle excision, and costoclavicular ligament division. The use of the endoscope enabled excellent visualization beneath the clavicle, allowing safe resection of the first rib posteriorly.^2,9,10)^ Given the patient’s tenderness beneath the clavicle in addition to chest pain, an endoscopic-assisted infraclavicular approach should be considered as one of the surgical options for decompressing the anterior elements of the thoracic outlet that cannot be addressed by conventional axillary or supraclavicular approaches.

When persistent chest pain remains unexplained after ruling out cardiovascular and respiratory conditions, TOS should be considered in the differential diagnosis. Physical examinations, including the Roos test, and CT imaging demonstrating subclavian stenosis with arm elevation can assist in diagnosing TOS.

## Conclusion

This case highlights neurogenic TOS presenting primarily as chest pain, which resolved following surgical intervention. Neurogenic TOS should be considered when chest pain persists despite excluding other potential causes.
